# Genetic Diversity of an Imperiled Neotropical Catfish and Recommendations for Its Restoration

**DOI:** 10.3389/fgene.2017.00196

**Published:** 2017-12-12

**Authors:** Fernando S. Fonseca, Rodrigo R. Domingues, Eric M. Hallerman, Alexandre W. S. Hilsdorf

**Affiliations:** ^1^Laboratório de Genética de Organismos Aquáticos e Aquicultura, Unidade de Biotecnologia, Núcleo Integrado de Biotecnologia, Universidade de Mogi das Cruzes, Mogi das Cruzes, Brazil; ^2^Instituto de Pesca, Agência Paulista de Tecnologia dos Agronegócios, Secretaria de Agricultura e Abastecimento do Estado de São Paulo, São José do Rio Preto, Brazil; ^3^Instituto do Mar, Universidade Federal de São Paulo, Santos, Brazil; ^4^Department of Fish and Wildlife Conservation, Virginia Polytechnic Institute and State University, Blacksburg, VA, United States

**Keywords:** *Steindachneridion parahybae*, surubim-do-paraíba, Paraíba do Sul River, Siluriformes, population structure, conservation aquaculture

## Abstract

The long-whiskered catfish *Steindachneridion parahybae* (Family Pimelodidae) is endemic to the Paraíba do Sul River basin in southeastern Brazil. This species was heavily exploited by artisanal fisheries and faces challenges posed by dams, introduced species, and deterioration of critical habitat. The remaining populations are small and extirpated from some locales, and the species is listed as critically endangered in Brazil. Screening variation at a partial mitochondrial control region sequence (mtCR) and 20 microsatellite loci, we: (i) describe the patterns of genetic diversity along its current distributional range; (ii) test the null hypothesis of panmixia; (iii) investigate the main factors driving its current population structure, and (iv) propose management of broodstock for fostering recovery of wild populations through genetically cognizant restocking. Our microsatellite data for 70 individuals from five collections indicate moderate levels of heterozygosity (*H*_O_ = 0.45) and low levels of inbreeding (*F*_IS_ = 0.016). Individual-based cluster analyses showed clear genetic structure, with three clusters of individuals over the collection area with no mis-assigned individuals, suggesting no recent migration among the three clusters. Pairwise *D*_EST_ values showed moderate and significant genetic differentiation among all populations so identified. The MUR population may have suffered a recent demographic reduction. mtCRs for 70 individuals exhibited 36 haplotypes resulting from 38 polymorphic sites. Overall, mitochondrial haplotype diversity was 0.930 (±0.023) and nucleotide diversity was 0.011 (±0.002). Significant population structure was observed, with ϕ_ST_ = 0.226. Genetic markers could be used in a hatchery-based restoration program emphasizing breeding of pairs with low kinship values in order to promote retention of genetic diversity and avoid inbreeding. Individual average kinship relationships showed 87.3% advised matings, 11.0% marginal matings, and 1.7% advised against. While these results comprise a contribution toward planning better breeding management and monitoring, parallel actions to be undertaken include surveying healthy riverine habits for reintroduction and continued searching for wild individuals to introduce new variation into the captive broodstock to avoid adaptation to captivity and to minimize inbreeding.

## Introduction

The Atlantic Forest is a globally important biome; with high freshwater fish diversity, a high degree of endemism, and threats of many impending extinctions, it is considered a conservation hotspot (Myers et al., 2000; Hubert and Renno, 2006). An important component of the fish fauna within this biome is the genus *Steindachneridion* (Order Siluriformes: Family Pimelodidae), which encompasses six recognized species of migratory long-whiskered catfishes that inhabit deep water in mid-sized streams with rocky bottoms (Agostinho et al., 2003; Garavello, 2005).

Commonly referred to “surubim-do-paraíba,” *Steindachneridion parahybae* (Steindachner, 1877) is a medium-sized, dorsoventrally flattened, whiskered catfish endemic to the Paraíba do Sul River basin in southeast Brazil (Garavello, 2005). This species was heavily exploited by artisanal fisheries along the Paraíba do Sul River in the early 1950s (Machado and Abreu, 1952). Records of historical harvest, however, are scarce, and survival of this species faces ongoing anthropogenic impacts (Hilsdorf and Petrere, 2002; Honji et al., 2009). Because of overfishing, construction of dams, introduced species, and deterioration of critical habitat (Loris, 2008; Moraes et al., 2017), the species is listed as “Critically Endangered” in the official lists of imperiled species in Brazil (Caneppele et al., 2008; Oyakawa et al., 2009).

Population decline is a prominent concern for conservation genetics because of potentially harmful consequences for species. Indeed, small and fragmented populations can undergo changes such as loss of genetic variation, fixation of deleterious alleles, inbreeding, and reduced fitness (Frankham et al., 2009). Critically, such outcomes can limit the ability of a given species to adapt to future environmental changes, increasing the risk of extinction (Frankham, 2005). Molecular markers are powerful tools for quantifying genetic variation at the individual, population, and species levels, and screening of markers can contribute to identification of units of relevance for the management and conservation of target species (Allendorf et al., 2010; Chauhan and Rajiv, 2010). Screening of molecular markers in studies of Neotropical freshwater fishes has contributed to assessment and conservation of fisheries genetic resources (Piorski et al., 2008; Hilsdorf and Hallerman, 2017). In particular, assessing genetic diversity and characterizing population structure is of primary importance for framing management strategies to minimize the likelihood of population extinction.

Once abundant in artisanal fisheries, *S. parahybae* is currently on the verge of extinction, with only a few remaining small and isolated populations (Honji et al., 2017). Management to foster recovery of the species is imperative due to its ecological importance as an apex predator and its economic importance for artisanal fisheries. Against this background, screening variation at a partial mitochondrial control region sequence (mtCR) and 20 microsatellite loci, we address for the first time issues important to the genetic conservation of the endemic Neotropical catfish *S. parahybae*. Specifically, we: (i) describe the patterns of genetic diversity of the *S. parahybae* along its current distributional range; (ii) test the null hypothesis of panmixia in this region; (iii) investigate the main factors driving its current population structure, and (iv) propose *ex situ* genetic breeding management for recovery of wild populations through genetically cognizant restocking programs.

## Materials and Methods

### Ethics Statement

All field work for fish sampling complied with the legal regulations of Brazil (collection permit SISBIO: 22140, Project: P&D CESP/ANEEL: 0061-017/2006).

### Sampling Sites and DNA Extraction

**FIGURE 1:**
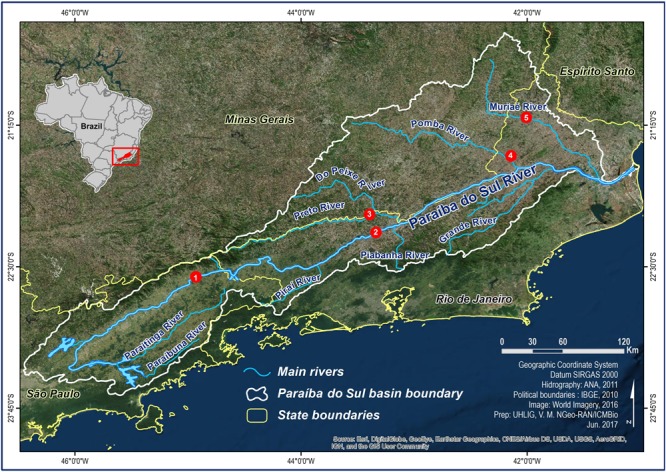
Sampling sites for *Steindachneridion parahybae* in the Paraíba do Sul River basin. 1 – Paraíba do Sul River – SP (PSP), 2 – Paraíba do Sul River – RJ (PRJ), 3 – Preto River - RJ (PRE), 4 – Pomba River – RJ (POM), 5 – Muriaé River – RJ (MUR).

A total of 70 individual *S. parahybae* were captured through an extensive occurrence survey along the tributaries of the Paraíba do Sul River basin in Brazil. Sites of occurrence were located with the help of local artisanal fishers and the Companhia Energética de São Paulo (São Paulo State Electric Power Company, CESP). Wild *S. parahybae* were collected at five different locations (**Figure 1**) within the Paraíba do Sul River basin in 2004 and between 2010 and 2015, consisting of: 1 individual from the Paraíba do Sul River (PSP) in São Paulo State (22°34′S; 44°53′W), 24 individuals from the Paraíba do Sul River (PRJ) in Rio de Janeiro State (22°13′S; 43°25′W), 5 individuals from the Preto River (PRE) in Rio de Janeiro State (22°00′S; 43°20′W), 3 individuals from the Pomba River (POM) in Rio de Janeiro State (21°32′S; 42°09′W), and 37 individuals from the Muriaé River (MUR) in Rio de Janeiro State (21°12′S; 41°55′W). All individuals were transferred live to aquaculture facilities belonging to CESP and kept as an *ex situ* germplasm bank (Supplementary Images S1, S2), where they were fin-clipped and individually marked with passive integrated transponder tags.

Total genomic DNA was extracted from approximately 20 mg of fin-clip tissue using a phenol-chloroform protocol adapted from Taggart et al. (1992) using STE (0.1 M NaCl, 0.05 M Tris-HCl, 0.01 M EDTA) with a lower concentration of EDTA buffer.

### Microsatellite Genotyping and Data Analysis

Genetic variability was screened at 20 di-, tri-, and tetra-nucleotide microsatellite loci (*Spa2, Spa3, Spa4, Spa5, Spa6, Spa7, Spa8, Spa11, Spa12, Spa14, Spa15, Spa16, Spa17, Spa18, Spa19, Spa20, Spa22, Spa23, Spa28*, and *Spa42*) described by Ojeda et al. (2016) using primer pairs developed by those authors. Sequences of all microsatellites were deposited in GenBank under accession numbers KU821557 to KU821572 and KX578067 to KX578070. Microsatellite alleles were genotyped using a 6.5% denatured polyacrylamide gel matrix (KB plus 6.5% Matrix Gel, Li-Color Biosciences, Lincoln, NE, United States) and a Li-Cor 4300 DNA Analyzer (IR2, Lincoln, NE, United States) using the IRDye^®^ 700 marker and universal M13 tail primer described by Schuelke (2000). The sizes of alleles were estimated by interpolating their position relative to molecular weight markers (50–350 bp DNA Sizing Standard IRDye^®^ 700) using the SagaGT Client program (Li-Cor Biosciences, Lincoln, NE, United States). All recommendations for minimizing genotyping errors originating from DNA quality and handling procedures (Pompanon et al., 2005) were implemented, including semi-automated scoring followed by visual inspection by two independent people.

The presence of null alleles and allelic dropout were tested for using MICRO-CHECKER 2.2.3 (Van Oosterhout et al., 2004). Deviations of genotype frequencies from Hardy–Weinberg expectations (HWE) were calculated by an exact test using the Markov chain randomization approach (Guo and Thompson, 1992) with HW-QUICKCHECK (Kalinowski, 2006). The significance for both types of tests was assessed employing a Markov chain Monte Carlo (MCMC) procedure based on 1,000 dememorizations, 100 batches, and 10,000 iterations per batch, and the critical value for significance was adjusted for multiple tests using the Holm–Bonferroni method (Holm, 1979). The nuclear diversity for each collection area and locus was quantified as the number of alleles per locus (*A*), allelic richness (*Ar*), observed heterozygosity (*H*_O_), expected heterozygosity (*H*_E_), and fixation index (*F*_IS_) using the Adegenet package (Jombart, 2008) in R v 3.1.2 (R Development Core Team, 2014). An individual assignment approach was performed using the Neighbor-Joining (NJ) algorithm using Euclidian distance between matrices of allele frequencies using the Adegenet package in R. In addition, discriminant analysis of principal components (DAPC, Jombart et al., 2010) was used to assign individuals to genetic cluster(s), using the Adegenet package in R. This multivariate approach was designed to identify and describe clusters of genetically related individuals in a manner similar to that performed by the Structure package (Pritchard et al., 2000). DAPC has the advantage that it does not require populations to be in Hardy–Weinberg equilibrium or linkage equilibrium between loci (Jombart et al., 2010). The optimal number of principal components retained followed the indication of α scores. The cluster assignments were pre-defined to correspond to *a priori* defined sample locations. Population genetic structure was estimated among clusters identified by DAPC using the population pairwise differentiation index *D*_EST_ (Jost, 2008) using the DEMETICS package in R (R Development Core Team, 2014). Holm–Bonferroni (Holm, 1979) adjustments of α were used to correct for multiple tests.

The program BOTTLENECK 1.2.02 (Cornuet and Luikart, 1997) was used to evaluate the possibility of changes in the recent effective size of a population. In this analysis, heterozygote excesses were checked using three different models: the infinite alleles model (IAM), stepwise mutation model (SMM), and the two-stage intermediate model (two-phase microsatellite evolution mode – TPM) with 70% SMM and 30% IAM. The two-phase model (TPM) is most powerful when fewer than 20 loci are used (Piry et al., 1999). The probability of significant heterozygosity excess was determined using 10,000 replications and a one-tailed Wilcoxon signed-rank tests (α = 0.05).

To guide design of matings for the restocking program, we performed average kinship assessment using the program COANCESTRY version 1.0.1.5 (Wang, 2011). This analysis is based on allele frequencies in the generation of matrices (*F*_0_) using means and variances of kinship coefficients. Three relationship categories (unrelated, half-sibling, and full-sibling) were used, with 5,000 pairs of simulated individuals for each category. In this evaluation, seven *r*_xy_ (KE) kinship estimators were used as follows: trioml (triadic likelihood estimator – Wang, 2007), wang (Wang, 2002), lynchLi (Li et al., 1993), lynchrd (Lynch and Ritland, 1999), ritland (Ritland, 1996), quellergt (Queller and Goodnight, 1989), and dyadml (dyadic likelihood estimator – Milligan, 2003). In addition, we applied the ML-Relate package (Kalinowski et al., 2006) to make a conservative estimation of the level of kinship. We then compared the results of both approaches.

### Mitochondrial DNA Amplification, Sequencing, and Data Analysis

A partial mitochondrial DNA control region (mtCR) was amplified by polymerase chain reaction (PCR) using two external primers, SPf (5′TCTAACTCCCAAAGCTAGAATC3′) and SPr (5′GGAACTTTCTAGGGTTCATCTTAAC3′), developed in this study. Amplification was performed in a 20-μl reaction containing: 20 ng/μl of genomic DNA, 0.5 IU *Taq* DNA polymerase (Thermo Fisher Scientific, Inc., São Paulo, Brazil), 1x buffer (100 mM Tris-HCl pH 8.8, 500 mM KCl), 1.5 mM MgCl_2_, 2.5 mM dNTPs, 10 mM of each primer, and ultrapure water. The PCR thermal-cycling profile consisted of initial denaturation at 94°C for 3 min; followed by 35 cycles of denaturation at 94°C for 1 min, annealing at 53°C for 1 min, and extension at 72°C for 1:30 min; and a final extension step at 72°C for 10 min.

The amplicons were purified using ExoSAP enzymes (Affymetrix, Cleveland, OH, United States) following the manufacturer’s instructions, except that the shortest time for deactivation of the enzymes was 15 min. The amplicons were sequenced on an Applied Biosystems 3130 Genetic Analyzer, and bases were called using Applied Biosystems Sequencing Analysis Software version 5.2. The sequences were edited manually when necessary using CodonCode (v.5.1.5, Codon Code Corporation, Centerville, MA, United States).

Summary statistics for genetic diversity – including haplotype (*Hd*) and nucleotide (π) diversities, number of haplotypes (*h*) and polymorphic sites (*S*) – were calculated in DnaSP v. 5.10 (Librado and Rozas, 2009). The relationships among haplotypes and their geographic distribution were assessed using the median-joining algorithm in NETWORK 4.6.1.1. To test the null hypothesis of matrilineal panmixia of *S. parahybae* along the Paraíba do Sul River basin, ϕ_ST_ (Weir and Cockerham, 1984) was calculated by analysis of molecular variance (AMOVA, Excoffier et al., 1992). To assess genetic structure across the collections, 10,000 permutations of the data were used to test the significance of hierarchical differentiation using Arlequin version 3.5.1.3 (Excoffier and Lischer, 2010).

The estimated probabilities were corrected using Holm–Bonferroni sequential adjustments for multiple tests (Holm, 1979). Due to small sample sizes at the PSP (*n* = 1), PRE (*n* = 5), and POM (*n* = 3) sites, data from those collection areas were included only to provide preliminary insights into how these collections may be related to the others.

Two neutrality metrics were calculated to infer the population demographic history of *S. parahybae, R*_2_ (Ramos-Onsins and Rozas, 2002) and *F*_S_ (Fu, 1997). Both metrics were calculated and their statistical significance tested using 10,000 permutations under the coalescent process implemented in DnaSP v.5.10 (Librado and Rozas, 2009).

## Results

### Microsatellite Intra- and Inter-population Diversity

All samples of *S. parahybae* from the five collection areas along the Paraíba do Sul River basin were successfully genotyped at 20 microsatellite loci. All loci within all collections were in Hardy–Weinberg equilibrium after correction for multiple tests. The linkage disequilibrium tests presented significant values after sequential Bonferroni correction (*p* < 0.05) for seven pairs of loci in a single population (MUR), which may be the consequence of many factors, including limited numbers of parents in the preceding generation, as well as natural selection, gene flow, assortative mating, and linkage. There was no evidence of genotyping errors, such as stuttering, null alleles, or large allele drop-out. The highest number of alleles (13) was amplified at the *Spa18* locus, while *Spa3, Spa7, Spa16, Spa23*, and *Spa28* all exhibited only two alleles. The number of alleles per locus within particular populations ranged between 2 and 13, with a mean of 4.7 ± 3.2. The allelic richness values ranged between 6.1 and 10.4, with average of 2.65 ± 2.07. The observed heterozygosity values ranged between 0.0 and 0.89, with average of 0.45 ± 0.24. Private alleles were observed in the MUR (14), PRE (1), and PRJ (2) collections. The *F*_IS_ inbreeding coefficient values ranged between -0.002 and 0.356, with average of 0.016. The summary statistics for all loci are shown in Supplementary Table S1.

**FIGURE 2:**
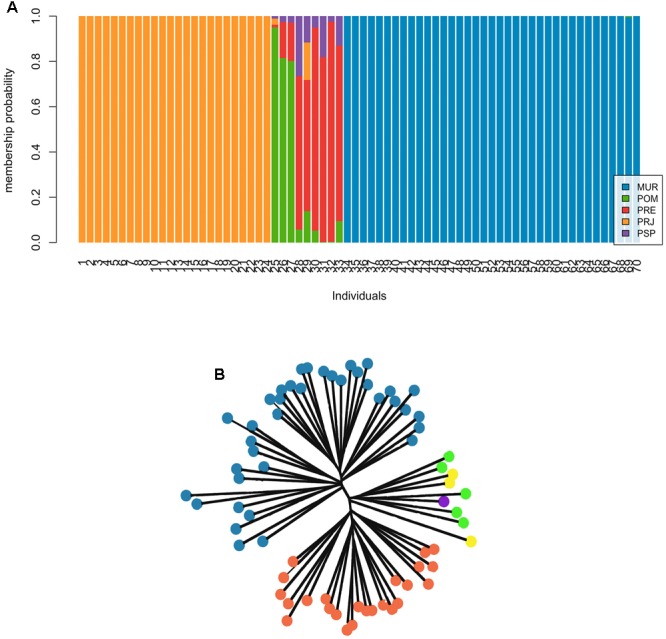
Different individual assignment approaches in *Steindachneridion parahybae*. **(A)** Results of the Bayesian approach with the use of discriminant analysis of principal components (DAPC) to investigate genetic structure. The ideal number of major components found for the analysis was seven based on the α scoring trend. Each vertical bar represents an individual in the DAPC diagram and each color represents the probability of belonging to one of the genetic groups. **(B)** Unrooted tree from a Neighbor-joining analysis of distance data.

Results of the individual-based DAPC and NJ cluster analyses showed clear genetic structure, with three clusters of individuals over the entire collection (**Figure 2**). Overall, individuals from the MUR and PRJ sites showed a 100% likelihood of assignment to their original collection area (hereafter termed populations). Individuals from POM, PSP, and PRE collections present a group of individuals of mixed ancestry (**Figure 2A**). Even though they do not form a same genetic cluster, we decided to assume a single cluster due to the low sample number. Neighbor-joining analysis (**Figure 2B**) also corroborated the three clusters of individuals: MUR, PRJ, and the POM/PSP/PRE sites. No mis-assigned individuals were observed, suggesting that there may have been no recent migration among these well-defined clusters.

The genetic differentiation indices were highly significant (*D*_EST_= 0.082, *p* = 0.001). The pairwise *D*_EST_ values for populations identified previously by NJ and DAPC analysis were congruent with the results of individual-based analysis, showing moderate and significant genetic differentiation among all populations (**Table 1**).

**Table 1:** Pairwise mtDNA *ϕ*_ST_ values (above diagonal) and pairwise Jost’s *D*_EST_ values using microsatellites (below the diagonal) among three population clusters of *Steindachneridion parahybae.*

Localities	PRJ	POM, PSP, and PRE	MUR
PRJ	–	0.1860^∗^	0.3044^∗^
POM, PSP, and PRE	0.0401^∗^	–	–0.0256^ns^
MUR	0.0688^∗^	0.0529^∗^	–

*^ns^Not significant, ^∗^highly significant (*p* < 0.01).*

*In silico* analyzes performed in BOTTLENECK 1.2.02 showed significant values (*p* < 0.05) for the MUR population using the SMM mutation model (**Table 2**). However, there were no significant values for TPM model, which is more powerful for fewer than 20 loci (Piry et al., 1999). These results suggest that among all populations, only the MUR population may have suffered recent demographic reduction. Because the POM, PSP, and PRE collections populations had few specimens, assessment of demographic bottlenecks in these populations had insufficient power.

**Table 2:** Results of the BOTTLENECK 1.2.02 program (Cornuet and Luikart, 1997) used to evaluate the possibility of changes in the effective size of the population.^1^

Localities	p IAM	*SD*	p SMM	*SD*	p TPM	*SD*
PRJ	0.387	0.075	0.074	0.034	0.344	0.448
POM	0.563	0.491	0.408	0.294	0.564	0.442
PRE	0.434	0.316	0.119	0.175	0.391	0.373
MUR	0.365	0.047	**0.034**	0.000	0.356	0.404

^1^*p is the probability value, with significant values (*p* < 0.05) shown in bold; IAM, the infinite alleles model; SMM, the stepwise mutation model; TPM, two-phase model of microsatellite evolution mode; and SD, standard deviation.*

### Mitochondrial Partial Control Region Sequence Variation

**FIGURE 3:**
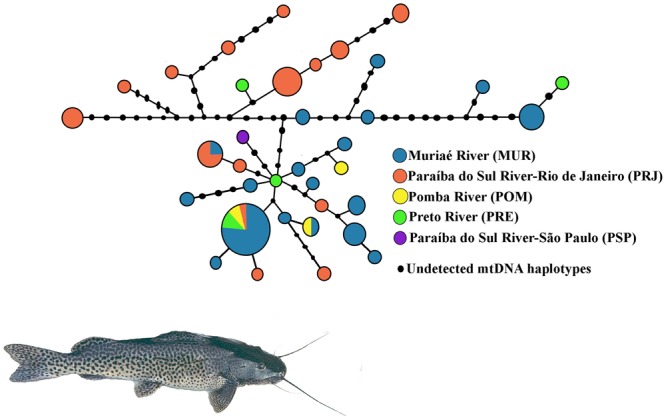
Unrooted haplotype network for mitochondrial D-loop sequences of *Steindachneridion parahybae* generated by NETWORK 4.1.1.2 based on the median-joining algorithm. The sizes of the respective circles are proportional to the frequencies of the haplotypes at issue.

A total of 791 bp of the mtCR were resolved from 70 *S. parahybae*. Sequences of all haplotypes were deposited in GenBank under accession numbers MG012754–MG012789. The sequences exhibited 36 haplotypes (Supplementary Tables S2, S3) resulting from 38 polymorphic sites and 41 mutations (34 transitions and 7 transversions). The nucleotide composition was 34.14% A, 19.66% C, 20.98% G, and 25.23% T. The overall *h* and π diversities were 0.930 (±0.023) and 0.011 (±0.002), respectively. The haplotype diversity values ranged from 0.868 ± 0.049 (MUR) to 0.99 ± 0.272 (POM), and nucleotide diversity values ranged from 0.005 ± 0.004 (POM) to 0.013 ± 0.007 (PRJ) (**Table 3**). Haplotype H2 (*n* = 13) was the most common, occurring mainly in MUR, and shared by all populations except for PSP (Supplementary Table S3). A total of 33 haplotypes were singletons, 16 occurring in MUR and 12 in PRJ. An unrooted median-joining tree of mtCR haplotypes (**Figure 3**) showed mainly singleton haplotypes in populations MUR and PRJ. Haplotype H2 was most frequent haplotype, shared by all *S. parahybae* populations except for PSP. Significant population structure was observed for *S. parahybae* along the Paraíba do Sul River basin based on mtCR data (ϕ_ST_ = 0.226, *p* < 0.05).

**Table 3:** Mitochondrial DNA sampling and descriptive statistics for *Steindachneridion parahybae*.

Sampling localities	*N*	*S*	*H*	*h ± SD*	π *± SD*	*F*_S_	*R*_2_
MUR	37	25	19	0.868 ± 0.049	0.008 ± 0.004	0.353^ns^	0.162^∗^
PRE	5	17	4	0.900 ± 0.161	0.011 ± 0.007	0.388^ns^	0.162^∗^
PRJ	244	34	14	0.935 ± 0.031	0.013 ± 0.007	0.377^ns^	0.163^∗^
PSP	1	0	1	–	–	–	–
POM	37	6	3	0.99 ± 0.272	0.005 ± 0.004	0.353^ns^	0.161^∗^

*(*N*) = number of sequenced individuals; (*S*) = number of polymorphic sites; *H* = haplotypes; (*h*) haplotype diversity; (π) nucleotide diversity; (SD) standard deviation; and *F*_*S*_ (Fu, 1997) and *R*_*2*_ (Ramos-Onsins and Rozas, 2002) indices of neutrality. ^ns^Not significant, ^∗^highly significant (*p* < 0.01).*

Fu’s *F_S_* metric presented high values, but there were no significant values (**Table 3**). In contrast, the *R*_2_ values were high and significant (*p* < 0.05), indicating a significant departure from neutrality. According to Ramos-Onsins and Rozas (2002), the *R*_2_ test is more powerful in detecting demographic events in small sample sizes, whereas Fu’s *F*_S_ is better for large sample sizes.

### Genetic Relatedness Among *Steindachneridion parahybae* in the *Ex Situ* Germplasm Bank

Among prospective broodstock in the *ex situ* germplasm bank, sampling variances for the kinship estimators (KE) ranged from 0.0198 to 0.1046. The lowest variance was found in the triadic likelihood estimator (TrioML). Considering that the KEs had high correlations and little influence on the inference of relatedness, paired kinship coefficients are reported only for the TrioML. Values estimated by the TrioML of kinship were low, indicating a low inbreeding index for 67 individuals, and thus, a range of permissible pairings among the broodstock candidates. Only three individuals with the highest values of inbreeding were classified as moderately endogamous. Means and variances for kinship likelihood estimators are showcased in Supplementary Table S4.

When we matched the 44 females with the 21 males among the broodstock candidates, we obtained 924 interactions that were added to the possible interactions with the five individuals of indeterminate sex (MUR12, MUR15, MUR20, MUR28, and MUR37), obtaining 1,269 possible matings. The index obtained for kinship values was split into three categories according to expected average theoretical values (Wang, 2011). That is, the relatedness values for simulated pairs were considered to be high above 0.5 (full-sib and parent-offspring), intermediate between 0.25 and 0.5 (half-sib or other kinship), and low below 0.25. Considering all possible matings, the calculated kinship relationships indicated 87.3% (1,108/1,269) advisable matings, 11.0% (140/1,269) marginal matings, and 1.7% (21/1,269) inadvisable matings. A table of consensus outcomes from the COANCESTRY and ML-Relate analyses is showcased in **Table 4**. Supplementary Tables S5, S6 show the results of the respective analyses.

**Table 4:** Consensus pairwise relatedness of broodstock candidates among the *Steindachneridion parahybae* individuals maintained in the germplasm bank, as assessed by both the COANCESTRY (TrioML estimator) and the ML-Relate packages.

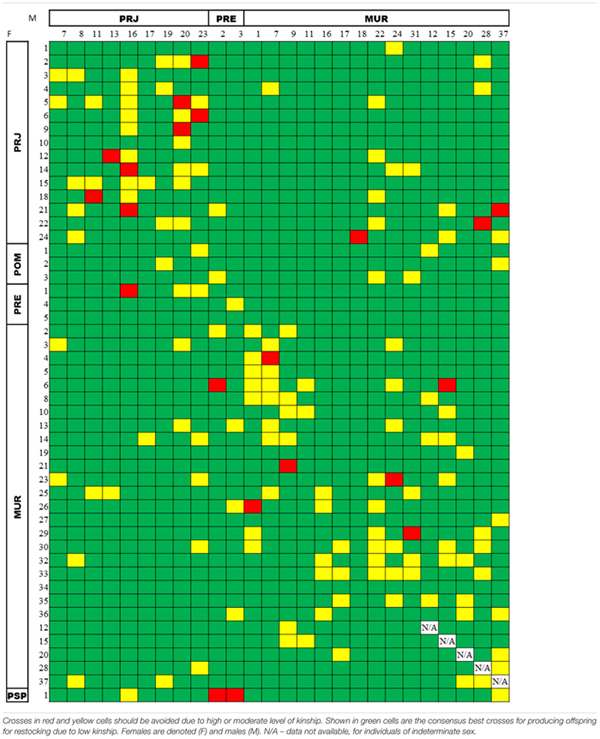

## Discussion

### Genetic Diversity and Population Structure

Our assessment of the extant *S. parahybae* populations reflected the endangered status of this Neotropical catfish in its area of occurrence. Attempts to locate wild populations proved the first indicator of the imperiled status of *S. parahybae* in the Paraíba do Sul watershed. Many efforts have been made during recent years to find and collect *S. parahybae* individuals to be kept in an *ex situ* gene bank so that a genetically cognizant restocking program (Miller and Kapuscinski, 2003) could be implemented. However, relatively few specimens have been found and collected until now. Although intensive efforts were made to collect specimens in localities where their occurrence was recorded by local anglers, at sites POM, PRE, and PSP, just a few individuals were collected; in the case of PSP, just one individual was collected in 2004. According to Honji et al. (2009), this species is regionally extinct in São Paulo State. At one collection site (PRJ), 24 individuals were collected in 2004 before an organochlorine insecticide (Endosulfan) spill into the Paraíba do Sul River killed fish and other riverine animals (Brito et al., 2012). After that, several unsuccessful attempts to capture *S. parahybae* have been made; hence, the species was likely extirpated at this site. The MUR is a locality where the species seems to be present, although in a small population. Despite the difficulty of catching this species in artisanal fisheries, local fishermen report the rare presence of *S. parahybae* in the Muriaé River.

Assessing the genetic differentiation of wild populations and individuals kept in *ex situ* gene banks is pivotal for developing conservation strategies to save a given species from extinction (FAO, 2008). Summary statistics for genetic diversity for the 20 microsatellite loci screened are shown in Supplementary Table S1. Heterozygosity (*H*_E_ = 0.47) and number of alleles per locus (*A* = 4.7) for wild *S. parahybae* populations were low across the 20 loci screened in this study.

Despite this low genetic variability, the inbreeding index (*F*_IS_ = 0.016) was below the average value (*F*_IS_ = 0.05) for 18 endangered animal species kept in *ex situ* conservation programs (Witzenberger and Hochkirch, 2011). The relatively low values of genetic diversity at microsatellite loci, a molecular marker type whose dynamics are more closely associated with contemporary events (Selkoe and Toonen, 2006; Weese et al., 2012), could be associated with the current state of fragility of populations likely impacted by anthropogenic habitat alterations and overfishing. These findings were corroborated with *in silico* analyses performed in BOTTLENECK 1.2.02.

The discreteness of populations of *S. parahybae* currently along the Paraíba do Sul River basin evident by analyses of microsatellite loci and mitochondrial DNA sequencing may be the result of isolation due to environmental alterations or to the characteristic short-distance reproductive migration behavior of this catfish species. No data are specifically available about the migration intensity of *S. parahybae*, but studies on catfishes in the Uruguay River, including the congeneric *S. inscripta*, show apparent short-distance migration (Zaniboni-Filho and Schulz, 2003). As the mobility of species is a factor determining the degree of homogenization of allele frequencies by gene flow (Avise, 2004), the contemporary genetic structure found for this species may then be a combination of short migration and river disruption by dams, which may act as barriers for gene flow (Dehais et al., 2010; Roberts et al., 2013; Gouskov et al., 2016). Indeed, this recent effect may be supported by the observed differentiation values for the nuclear markers, indicating low to moderate differentiation within the contemporary period.

The differentiation shown for mitochondrial D-loop sequences (ϕ_ST_ = 0.226) and microsatellites (*D*_EST_ = 0.082) can be interpreted as reflecting historical and recent structuring, especially for the female component of the population, with some degree of homogenizing gene flow for males, with or without the effects of recent anthropogenic isolation. Cluster analyses (**Figure 2**) showed considerable population structure, indicating the division of the *S. parahybae* gene pool into three distinct groups, MUR, PRJ, and a mixed group formed by POM, PSP, and PRE. The strongly supported assigned clusters for MUR and PRJ (*q* > 0.9) denoted virtual absence of migration between the two groups. The identification and characterization of an Evolutionary Significant Unit (ESU), based on population genetic structure and adaptive differentiation, would provide a useful basis for conservation, as well as a more complete understanding of the role of adaptive evolution in the natural history of the species (Casacci et al., 2014). Carvalho et al. (2012) reported two ESUs within the widely distributed *Pseudoplatystoma corruscans*, a commercially important Neotropical siluriform catfish. However, despite the presence of private alleles in the MUR sample, populations of *S. parahybae* may more appropriately be considered Management Units (MUs, Moritz, 1994), which can be defined as populations with significant divergence of allele frequencies at nuclear or mitochondrial loci, regardless of the phylogenetic distinctiveness of the alleles. Taking another approach, Palsbøll et al. (2007) proposed that MUs should be determined by the level of interpopulation genetic divergence in general; however, the threshold values to establish populations’ genetic connectivity must be flexible to reflect each specific biological and conservation context. This is particularly the case for the Muriaé River population, where *S. parahybae* individuals yet seem to occur.

### Phylogeography and Demographic History

Despite the difficulty of comparing among studies the average divergence between mtDNA sequences due to lack of standardization of sequence length, small nucleotide diversity combined with considerable haplotype diversity (Grant and Bowen, 1998) might suggest a demographic expansion after a period of low effective population size for *S. parahybae* populations. Mesquita et al. (2001) assessed *Chondrostoma lusitanicumem*, an endangered fish species in Portugal, and found the same combination of molecular genetic variation among marker types, suggesting that high mitochondrial haplotype diversity with low nucleotide divergence may indicate non-equilibrium evolutionary action or non-neutral forces such as “founder-flush” population differentiation or even the persistence of slightly deleterious mutations in small populations due to ineffectiveness of “purifying” selection. In the present study, the hypothesis of demographic expansion is supported by results of the *R_2_* test, a test to detect population growth. The positive, significant values refuted the null hypothesis of constant population size under the neutral model. In addition, the haplotype network (**Figure 3**) does not have the star-like shape typical of populations that show a signature of a species that has expanded its numbers from a modest number of founders (Avise, 2000). It is likely that throughout the evolutionary history of *S. parahybae*, different mitochondrial lineages colonized and developed in each locality of the basin, and that carriers of distinct mitochondrial lineages migrated along the basin when no artificial barriers, such as dams, were present. Such dams are barriers that prevent the reproductive migration of many fish species (Pelicice et al., 2015). As human occupation expanded along the watershed, overfishing, pollution, introduction of species, and dam construction for electric power generation impacted *S. parahybae* population size and geographic range, accompanied by the local and global loss of many mitochondrial haplotypes. For instance, the only specimen captured from PSP has a unique haplotype at the terminus of a branch of the network, not present in any other sampling site in this survey. In addition, the existence of many different mitochondrial haplotypes in the MUR and PRJ populations, many separated by inferred mutational differences not represented by observations in living individuals, supports this interpretation.

On the other hand, the result of the BOTTLENECK test suggests that the MUR population was subject to a recent population bottleneck. This result is supported by observation of excess heterozygosity in the population as a whole. The bottleneck process, which the extant *S. parahybae* population may have undergone, suggests the loss of rare alleles during the population reduction. The loss of alleles may ultimately jeopardize the population due to loss genetic variability crucial for meeting future ecological challenges; however, the presence of private alleles may also suggest local adaptation (Santos et al., 2016). The absence of *S. parahybae* individuals in the artisanal fisheries in localities where they were common as recently as the early 1950s (Machado and Abreu, 1952) and in scientific samples at sites of previous occurrence in the Paraíba do Sul River basin clearly suggests that *S. parahybae* populations underwent a genetic bottleneck process and that some populations were extirpated.

### A Restoration Genetics Approach to Recovery of *S. parahybae* Populations

Restocking of freshwater fishes is often conducted to restore populations impacted by the construction of hydroelectric power dams (Marmulla, 2001; Palmer et al., 2007; Piorski et al., 2008). While the effectiveness of the approach has been questioned (Snyder et al., 1996; Fraser, 2008; Neff et al., 2011), restoration of imperiled or extirpated populations must be considered when other efforts have proven unsuccessful (Attard et al., 2016). Restoration genetics attempts to use the tools of genomics to support proper management of captive populations and propagation of genetically healthy fingerlings (Frankham, 2010). Human-driven environmental changes in the Paraíba do Sul watershed date to the onset of agricultural production (sugarcane in the 17th century, coffee in the late 18th–19th centuries), and industrialization from the 1950s (Pádua, 2017). More than 120 hydropower stations in the Paraíba do Sul River and its tributaries have disrupted fluvial connectivity, leading to losses of local populations and species (Agostinho et al., 2008; Loris, 2008). *Steindachneridion parahybae* is among the endemic fishes of Paraíba do Sul negatively impacted by human actions, and is listed as threatened by the environmental agencies of the Brazilian government (Honji et al., 2017). The recovery of these imperiled species has been promoted by establishing *ex situ* germplasm banks and genetics-based captive breeding programs. The reintroduction programs for *S. parahybae* follow to some degree the framework proposed by Attard et al. (2016), which accounts for past, present, and future components of captive-breeding and reintroduction. The geographical localization and genetic characterization of extant *S. parahybae* populations described herein, and their tagging and maintenance in the *ex situ* gene bank facilities encompass the past and present components of the holistic framework for successful reintroductions.

Successful captive breeding depends upon keeping levels of inbreeding low and optimizing genetic variability so that fingerlings intended for reintroduction reflect as much as possible the genetic makeup of the species. To achieve this goal, we assessed the genetic relatedness of all captively bred individuals to generate guidance for the hatchery manager to select genetically unrelated fish to be crossed (Saura et al., 2013). A set of 20 species-specific microsatellite markers (Ojeda et al., 2016) was used successfully to assess the mean kinship within and among the remaining populations of *S. parahybae*, generating relatedness indices between each captive individual. Keeping the minimum number of founders to minimize a loss of genetic diversity, avoiding both inbreeding and outbreeding depression, and searching for new wild populations to constantly renew the captive stock are all pivotal to the success of *ex situ* conservation projects (Miller and Kapuscinski, 2003; Witzenberger and Hochkirch, 2011; IUCN/SSC, 2013). Neff et al. (2011) argued that just taking into account multilocus heterozygosity to measure the likely effectiveness of reintroduction is too simplistic. According to these authors, it is more important to come to better comprehend the complexity of the genetic architecture of fitness, so that the importance of genetic diversity to the evolutionary potential of populations of fishes and their subsequent adaptation after reintroduction can be gauged. Therefore, they recommended that effective management and conservation of populations in imminent peril of extirpation should include: (i) rehabilitation and maintenance of habitat and ecological function; (ii) incorporation of natural ecological processes into artificial breeding programs, and (iii) use of a full or partial factorial breeding design in artificial breeding programs. This last recommendation regarding breeding design is intended to maximize the amount of genetic differentiation throughout the species’ occurrence.

Use of estimators for pairwise relatedness and individual inbreeding coefficients based on multilocus microsatellite markers has proven an efficient strategy to achieve better responses in reintroduction programs. Sriphairoj et al. (2007), working with the critically endangered Mekong giant catfish *Pangasianodon gigas* tested different scenarios regarding different broodstock recruitment and mating strategies. The authors suggested that using minimal kinship in a random mating scheme could keep the effective population size (*N*_e_) at 100 so that allelic diversity can be retained at above 90% of current allelic diversity for at least four generations. In the present study of endangered *S. parahybae* populations, we used this same approach, also based on multilocus microsatellite markers, to genetically characterize the few remaining wild populations and broodstock currently maintained in an *ex situ* germplasm bank. These genetic data comprise a contribution toward planning better breeding management and monitoring any changes in the genetic makeup of the broodstock, as well as in the reintroduced fingerlings in their new environments. Parallel actions to be taken would include surveying healthy riverine habits for reintroduction and continued searching for wild individuals to introduce new variation into the captive broodstock to avoid adaptation to captivity and to prevent inbreeding. Successes in other genetic marker-assisted restoration programs suggest that application of this approach might also prove effective for *S. parahybae*. Olsen et al. (2000) used microsatellite DNA markers to identify a targeted stock of pink salmon *Oncorhynchus gorbuscha* in a supportive breeding program on the Dungeness River in Washington State, United States, providing the basis for hatchery-based supplementation. Following cessation of stocking from outside sources, microsatellite marker-assisted hatchery-based supplementation of walleye *Sander vitreus* (Palmer et al., 2007) contributed to a rebuilding of the native population in the New River, Virginia, United States and its recognition as a premier recreational fishery for the species.

## Author Contributions

AH conceived and supervised the project. FF performed the experiments, analyzed data, and drafted the manuscript, RD performed statistical analyses and contributed to manuscript writing, AH and EH wrote and critically edited the final manuscript. All authors read and approved the final version of the manuscript.

## Conflict of Interest Statement

The authors declare that the research was conducted in the absence of any commercial or financial relationships that could be construed as a potential conflict of interest.
