# Supplementary material for: Genetic Diversity of an Imperiled Neotropical Catfish and Recommendations for Its Restoration
Source: Front Genet. 2017 Dec 12;8:196. doi: 10.3389/fgene.2017.00196 (PMC5732928; doi:10.3389/fgene.2017.00196)
Supplement: Supplementary file 1 [file Supplementary_Material_Tables.pdf]

SUPPLEMENTARY MATERIAL – TABLES

Genetic diversity of an imperiled Neotropical catfish and recommendations for its restoration

Fernando Stopato da Fonseca, Rodrigo Rodrigues Domingues, Eric M. Hallerman and Alexandre Wagner Silva Hilsdorf

Table S1. Summary statistics for genetic diversity at 20 microsatellite loci of *Steindachneridion parahybae*: (A) number of alleles; (Ar) allelic richness; (H_O) observed heterozygosity; (H_E) expected heterozygosity; (F_{IS}) inbreeding coefficient; (NR) not reported; (PIC) Polymorphism Information Content; (Acc. N.) Genebank Accession Number; *Hardy-Weinberg equilibrium significance (P < 0.05).

Locus		MUR	PRE	PRJ	POM
Spa2	***A***	8	6	6	4
PIC	***Ar***	6.101	4.167	4.099	3.000
0.809	H_O	0.613*	1.000	0.875	1.000
Acc. N.	H_E	0.836	0.760	0.756	0.667
KU821557	F_{IS}	0.267	-0.316	-0.157	-0.500
	Private alleles	2	0	0	0
Spa3	***A***	2	1	2	1
PIC	***Ar***	1.144	1.000	1.133	1.000
0.102	H_O	0.135	0.000	0.125	0.000
Acc. N.	H_E	0.126	0.000	0.117	0.000
KU821558	F_{IS}	-0.072	NR	-0.067	NR
	Private alleles	0	0	0	0
Spa4	***A***	4	3	3	3
PIC	***Ar***	1.157	1.515	1.825	2.000
0.294	H_O	0.143	0.400	0.375	0.667
Acc. N.	H_E	0.136	0.340	0.452	0.500
KU821559	F_{IS}	-0.051	-0.176	0.171	-0.333
	Private alleles	0	0	0	0
Spa5	***A***	3	3	3	3
PIC	***Ar***	2.460	1.852	2.268	2.571
0.510	H_O	0.594*	0.600	0.667	0.333
Acc. N.	H_E	0.593	0.460	0.559	0.611
KU821560	F_{IS}	-0.002	-0.304	-0.192	0.454
	Private alleles	0	0	0	0
Spa6	***A***	4	3	3	3
PIC	***Ar***	2.284	2.174	2.203	2.571
0.493	H_O	0.444	0.600	0.542	1.000
Acc. N.	H_E	0.562	0.540	0.546	0.611
KU821561	F_{IS}	0.209	-0.111	0.008	-0.636
	Private alleles	1	0	0	0
Spa7	***A***	2	2	2	2
PIC	***Ar***	1.993	2.000	1.8	2.000
0.373	H_O	0.543	0.600	0.583	0.333
Acc. N.	H_E	0.498	0.500	0.444	0.500
KU821562	F_{IS}	-0.089	-0.200	-0.312	0.333
	Private alleles	0	0	0	0
Spa8	***A***	4	3	4	4
PIC	***Ar***	2.928	2.631	3.122	3.600
0.643	H_O	0.676	1.000	0.667	1.000
Acc. N.	H_E	0.658	0.620	0.679	0.722
KU821563	F_{IS}	-0.026	-0.613	0.019	-0.384
	Private alleles	0	0	0	0

Spa11	***A***	4	2	3	1
PIC	***Ar***	1.116	1.470	1.453	1.000
0.186	H_O	0.108	0.400	0.375	0.000
Acc. N.	H_E	0.104	0.320	0.311	0.000
KU821564	F_{IS}	-0.038	-0.250	-0.203	NR
	Private alleles	1	0	0	0
Spa12	***A***	7	6	7	3
PIC	***Ar***	5.705	4.167	3.892	2.571
0.782	H_O	0.656	1.000	0.792	0.667
Acc. N.	H_E	0.825	0.760	0.743	0.611
KU821565	F_{IS}	0.204	-0.316	-0.065	-0.091
	Private alleles	2	0	1	0
Spa14	***A***	5	2	4	5
PIC	***Ar***	2.074	1.724	1.855	4.500
0.472	H_O	0.567	0.600	0.500	1.000
Acc. N.	H_E	0.518	0.420	0.461	0.778
KX578067	F_{IS}	-0.096	-0.428	-0.087	-0.286
	Private alleles	0	0	0	0
Spa15	***A***	3	3	3	3
PIC	***Ar***	2.228	2.941	1.184	2.000
0.420	H_O	0.486	0.600	0.167	0.667
Acc. N.	H_E	0.551	0.660	0.155	0.500
KU821566	F_{IS}	0.117	0.091	-0.072	-0.333
	Private alleles	0	0	0	0
Spa16	***A***	2	2	2	2
PIC	***Ar***	1.685	1.724	1.800	1.384
0.332	H_O	0.297	0.600	0.583	0.333
Acc. N.	H_E	0.406	0.420	0.444	0.278
KU821567	F_{IS}	0.268	-0.428	-0.312	-0.200
	Private alleles	0	0	0	0
Spa17	***A***	8	4	7	4
PIC	***Ar***	3.705	2.941	3.622	3.600
0.724	H_O	0.621	0.800	0.875	1.000
Acc. N.	H_E	0.730	0.660	0.724	0.722
KU821568	F_{IS}	0.148	-0.212	-0.208	-0.384
	Private alleles	1	0	1	0
Spa18	***A***	13	5	10	2
PIC	***Ar***	10.453	3.846	6.811	1.600
0.883	H_O	0.857	0.600	0.895	0.500
Acc. N.	H_E	0.904	0.740	0.853	0.375
KU821569	F_{IS}	0.052	0.189	-0.049	-0.333
	Private alleles	3	0	0	0
Spa19	***A***	2	2	2	2
PIC	***Ar***	1.219	1.219	1.087	1.384
0.156	H_O	0.200	0.200	0.083	0.333
Acc. N.	H_E	0.180	0.180	0.080	0.278
KU821570	F_{IS}	-0.111	-0.111	-0.043	-0.200
	Private alleles	0	0	0	0
Spa20	***A***	3	2	3	2
PIC	***Ar***	2.040	1.219	1.542	1.384
0.383	H_O	0.600*	0.200	0.250 *	0.333
Acc. N.	H_E	0.510	0.180	0.351	0.278
KX578068	F_{IS}	-0.177	-0.111	0.289	-0.200
	Private alleles	0	0	0	0
Spa22	***A***	2	1	2	2
PIC	***Ar***	1.508	1.000	1.229	1.800
0.262	H_O	0.428	0.000	0.208	0.000
Acc. N.	H_E	0.337	0.000	0.187	0.444
KU821571	F_{IS}	-0.273	NR	-0.116	1.000
	Private alleles	1	0	0	0
Spa23	***A***	2	2	2	1
PIC	***Ar***	1.144	1.470	1.087	1.000
0.113	H_O	0.081	0.400	0.083	0.000

Acc. N.	***H_E***	0.126	0.320	0.080	0.000
KU821572	***F_{IS}***	0.356	-0.250	-0.043	NR
	Private alleles	0	0	0	0
Spa28	***A***	2	1	1	1
PIC	***Ar***	1.314	1.000	1.000	1.000
0.125	***H_O***	0.222	0.000	0.000	0.000
Acc. N.	***H_E***	0.239	0.000	0.000	0.000
KX578069	***F_{IS}***	0.071	NR	NR	NR
	Private alleles	1	0	0	0
Spa42	***A***	8	1	5	4
PIC	***Ar***	2.297	1.000	2.127	4.000
0.537	***H_O***	0.606	0.000	0.400	1.000
Acc. N.	***H_E***	0.565	0.000	0.530	0.750
KX578070	***F_{IS}***	-0.073	NR	0.245	-0.333
	Private alleles	2	1	0	0
Average	***A***	4.4	2.7	3.7	2.6
	Ar	2.727	2.053	2.256	2.198
	H_O	0.443	0.480	0.452	0.508
	H_E	0.470	0.394	0.423	0.431
	F_{IS}	0.034	-0.221	-0.063	-0.151
	Private alleles	14	1	2	0

Table S2. Summary of nucleotide variation found in the partial mitochondrial DNA control region sequenced.

	Nucleotide site																																										
**Haplotypes**	**6**	**13**	**27**	**28**	**79**	**110**	**114**	**115**	**162**	**183**	**203**	**211**	**244**	**253**	**254**	**277**	**285**	**293**	**346**	**364**	**372**	**374**	**384**	**410**	**436**	**492**	**494**	**505**	**506**	**507**	**533**	**534**	**544**	**585**	**594**	**605**	**635**	**636**	**642**	**643**	**655**	**763**	**772**
**MG012755**	A	A	A	G	A	.	G	G	T	T	C	C	G	A	C	A	G	G	T	T	C	T	C	A	T	C	A	G	T	A	C	G	A	A	T	C	C	T	C	-	A	A	A
**MG012789**	A	A	A	C	.	C	.	A	.	.	.	.	A	.	.	.	.	.	.	.	.	.	.	.	.	.	.	.	.	.	.	A	.	.	.	.	.	.	.	-	.	.	G
**MG012788**	.	.	.	.	.	.	.	A	.	.	.	.	.	.	.	.	.	.	.	.	.	.	.	.	.	.	.	.	.	.	.	G	.	.	.	.	T	.	.	C	.	.	G
**MG012787**	.	G	.	A	.	.	A	A	.	C	.	.	A	.	.	.	.	.	.	.	.	.	.	.	.	T	G	.	.	.	.	.	G	.	C	.	T	.	-	-	.	-	G
**MG012786**	.	.	.	.	.	.	.	A	.	.	.	.	A	.	.	.	.	.	C	.	.	.	.	.	.	T	G	.	C	G	.	.	G	.	.	.	T	C	.	C	.	.	G
**MG012785**	.	.	.	.	.	.	A	A	.	.	.	.	A	.	.	G	.	.	.	.	A	.	.	.	.	T	G	.	C	.	.	.	G	G	.	.	T	.	.	-	.	.	G
**MG012784**	.	.	.	.	T	.	A	A	.	C	.	.	A	.	.	.	.	.	.	.	.	.	.	.	.	.	G	.	.	.	.	.	G	.	C	.	T	.	.	C	.	-	G
**MG012783**	.	.	.	.	.	.	A	A	.	.	.	.	A	.	.	G	.	.	.	.	A	.	.	.	.	T	G	.	C	.	.	.	G	.	.	.	T	.	.	-	.	.	G
**MG012782**	.	.	.	.	.	C	.	A	.	.	.	.	.	.	T	.	.	.	.	.	.	.	.	.	.	.	.	.	.	.	.	.	.	.	.	.	.	.	.	-	.	.	G
**MG012781**	.	.	G	A	.	.	A	A	C	.	.	.	A	.	.	.	.	.	.	.	.	C	.	G	.	.	G	A	C	G	.	.	G	.	.	.	T	.	.	C	.	.	G
**MG012780**	.	G	.	A	.	.	A	A	.	C	.	.	A	.	.	.	.	.	.	.	.	.	.	.	.	T	G	.	.	.	.	.	G	.	C	-	T	.	.	C	.	-	G
**MG012779**	.	.	.	.	.	.	.	.	.	.	.	.	.	.	.	.	.	.	.	.	.	.	.	.	.	T	G	.	C	.	.	.	G	.	.	.	.	.	.	-	.	.	G
**MG012778**	G	.	.	.	.	.	A	A	.	.	.	.	A	.	.	G	.	.	.	.	A	.	.	.	.	T	G	.	C	.	.	.	G	.	.	.	T	.	.	C	G	.	G
**MG012777**	.	.	.	.	.	.	A	A	.	C	.	.	A	.	.	G	.	.	.	.	A	.	.	.	.	T	G	.	C	.	.	.	G	G	.	.	T	.	.	-	.	.	G
**MG012776**	.	.	.	.	.	.	.	A	.	.	.	.	.	.	.	G	.	.	.	.	.	.	.	.	.	.	.	.	.	.	.	G	.	.	.	.	.	.	-	-	.	.	G
**MG012775**	.	.	.	.	.	.	.	A	.	.	.	.	.	.	.	.	.	.	.	.	.	.	.	.	.	.	.	.	.	.	.	G	.	.	.	.	.	.	.	-	.	.	G
**MG012774**	.	.	.	.	.	.	A	A	.	C	.	.	A	.	.	G	.	.	.	.	A	.	.	.	.	.	G	.	C	.	.	G	G	.	.	.	T	.	.	-	.	.	G
**MG012773**	.	.	.	.	.	.	C	A	.	.	.	.	A	G	.	G	.	A	.	C	.	C	T	.	.	.	G	T	C	.	.	G	G	.	.	.	T	.	.	-	.	.	G
**MG012772**	.	.	.	.	.	.	.	A	.	.	.	.	.	.	.	.	A	.	.	.	.	.	.	.	.	.	.	.	.	.	.	G	.	.	.	.	.	.	.	-	.	.	G
**MG012771**	.	.	.	.	.	.	.	A	.	.	.	.	A	.	.	.	A	.	.	.	.	.	.	.	.	.	.	.	.	.	.	.	G	.	.	.	T	.	.	-	.	.	G
**MG012770**	.	.	.	C	.	.	.	A	.	.	.	.	.	.	.	.	.	.	.	.	.	.	.	.	.	.	.	.	.	.	.	G	.	.	.	.	.	.	.	-	.	.	G
**MG012769**	.	.	.	.	.	.	.	.	.	.	.	.	.	.	.	.	.	.	.	.	.	.	.	.	.	.	.	.	.	.	.	G	G	.	.	.	.	.	.	-	.	.	G
**MG012768**	.	.	.	.	.	.	.	A	.	.	.	.	.	.	.	G	.	.	.	.	.	.	.	.	.	.	.	.	.	.	.	G	.	.	.	.	.	.	-	-	.	.	.
**MG012767**	.	.	.	.	.	.	.	.	.	.	.	.	.	.	.	.	.	.	.	.	.	.	.	.	A	.	.	.	.	.	.	G	.	.	.	.	.	.	.	-	.	.	.
**MG012766**	.	.	.	.	.	.	C	A	.	.	.	.	A	G	.	.	.	.	.	.	.	.	.	.	.	.	.	.	C	.	.	G	.	.	.	.	.	.	.	-	.	.	G
**MG012765**	.	.	.	.	.	.	.	.	.	.	.	.	.	.	.	.	.	.	.	.	.	.	.	.	.	.	.	.	.	.	T	G	.	.	.	.	.	.	.	-	.	.	.
**MG012764**	.	.	.	.	.	.	.	A	.	.	.	.	.	.	T	.	.	.	.	.	.	.	.	.	.	.	.	.	.	.	T	G	.	.	.	.	.	.	.	-	.	.	G
**MG012763**	.	.	.	.	.	.	C	A	.	.	A	.	A	.	T	G	.	.	.	.	.	.	.	.	.	.	.	.	.	.	.	G	.	.	.	.	.	.	.	-	.	.	G
**MG012762**	.	.	.	.	.	.	C	A	.	.	.	.	.	.	.	.	.	.	.	.	.	.	.	.	.	.	.	.	C	.	.	G	G	.	.	.	T	.	.	-	.	.	G
**MG012761**	.	.	.	.	.	.	.	.	.	.	.	.	.	.	.	.	.	.	.	.	.	.	.	.	.	.	.	.	.	.	.	G	.	.	.	.	.	.	.	-	.	.	G
**MG012760**	.	.	.	C	.	C	.	A	.	.	.	.	.	G	T	.	.	.	.	.	.	.	.	.	.	.	.	.	.	.	.	G	.	.	.	.	.	.	.	-	.	.	G
**MG012759**	.	.	.	.	.	.	C	A	.	.	A	T	A	G	.	G	.	A	.	C	.	C	T	.	.	.	.	.	.	.	.	.	.	.	.	.	.	.	.	-	.	.	G
**MG012758**	.	.	.	C	.	C	.	A	.	.	.	.	.	G	T	.	.	.	.	.	.	.	.	.	.	.	.	.	.	.	.	.	.	.	.	.	.	.	.	-	.	.	G
**MG012757**	.	.	.	.	.	.	.	A	.	.	.	.	.	G	T	.	.	.	.	.	.	.	.	.	.	.	.	.	.	.	.	.	.	.	.	.	.	.	.	-	.	.	G
**MG012756**	.	.	.	.	.	.	C	A	.	.	.	.	A	G	.	.	.	.	.	.	.	.	.	.	.	.	.	.	C	.	.	G	.	.	.	.	.	.	.	-	.	.	G
**MG012754**	.	.	.	.	.	.	C	A	.	.	A	T	A	G	.	G	.	A	.	C	.	C	T	.	.	.	G	T	C	.	.	G	G	.	.	.	T	.	.	-	.	.	G

Table S3. Distribution of *Steindachneridion parahybae* mtDNA haplotypes among populations sampled across the Paraíba do Sul watershed.

Haplotypes (H)	MUR (37)	PRE (5)	PRJ (24)	PSP (1)	POM (3)
H1	4*	0	0	0	0
H2	13	2	1	0	1
H3	1*	0	0	0	0
H4	2*	0	0	0	0
H5	3*	0	0	0	0
H6	1*	0	0	0	0
H7	1*	0	0	0	0
H8	1	0	0	0	1
H9	1*	0	0	0	0
H10	1*	0	0	0	0
H11	1*	0	0	0	0
H12	1*	0	0	0	0
H13	1*	0	0	0	0
H14	1*	0	0	0	0
H15	1	0	3	0	0
H16	1*	0	0	0	0
H17	1*	0	0	0	0
H18	1*	0	0	0	0
H19	1*	0	0	0	0
H20	0	1*	0	0	0
H21	0	1*	0	0	0
H22	0	1*	0	0	0
H23	0	0	1*	0	0
H24	0	0	5*	0	0
H25	0	0	2*	0	0
H26	0	0	1*	0	0
H27	0	0	1*	0	0
H28	0	0	3*	0	0
H29	0	0	1*	0	0
H30	0	0	2*	0	0
H31	0	0	1*	0	0
H32	0	0	1*	0	0
H33	0	0	1*	0	0
H34	0	0	1*	0	0
H35	0	0	0	1*	0
H36	0	0	0	0	1*

Exclusive haplotypes are indicated with *.

Table S4. Means and variances for kinship likelihood estimators implemented in the program COANCESTRY.

	TrioML	Wang	LynchLi	LynchRd	Ritland	QuellerGt	DyadML
Mean	0.11234	-0.12147	-0.11165	-0.03103	-0.0285	-0.0489	0.14476
Variance	0.01981	0.10317	0.10457	0.04343	0.06944	0.7922	0.02732

Table S5. Pairwise relatedness of broodstock candidates among the *Steindachneridion parahybae* individuals maintained in the germplasm bank, assessed by the TrioML estimator (Coancestry)

	M	PRJ									PRE		MUR														
F		7	8	11	13	16	17	19	20	23	2	3	1	7	9	11	16	17	18	22	24	31	12	15	20	28	37
PRJ	1	5	0	4	19	0	12	23	4	4	4	3	12	0	0	24	0	0	2	0	28	19	0	0	6	7	9
	2	0	27	0	7	11	1	33	26	42	8	5	0	8	1	0	0	0	0	1	0	21	0	0	9	26	3
	3	15	43	0	2	35	2	0	1	0	0	4	0	4	9	4	1	0	0	0	6	9	0	6	1	0	5
	4	0	0	0	18	37	5	31	4	7	2	8	0	15	16	0	0	3	0	0	0	0	0	7	4	12	1
	5	27	0	2	5	31	11	11	59	30	4	0	0	0	0	0	0	0	4	24	0	0	0	0	0	3	5
	6	0	1	13	3	28	2	7	36	52	0	5	0	4	0	1	1	5	0	15	0	5	1	0	0	1	3
	9	4	7	0	22	29	2	4	49	0	11	2	0	0	0	0	6	8	0	8	3	0	0	0	1	1	4
	10	5	0	14	5	24	0	2	31	0	17	19	0	0	0	1	1	6	0	0	5	1	10	0	3	1	4
	12	0	4	15	51	15	8	0	6	5	0	0	0	0	0	0	0	0	8	25	0	11	0	0	4	7	0
	14	0	0	5	1	49	5	0	15	19	12	20	0	16	0	0	0	0	0	1	46	15	0	0	0	0	0
	15	0	2	17	2	28	25	0	35	4	0	6	0	7	0	1	0	10	0	8	0	0	0	0	3	7	4
	18	5	0	49	7	14	9	1	0	20	3	0	6	0	0	0	0	0	5	9	0	9	0	3	5	11	9
	21	0	43	0	5	50	7	2	9	12	33	3	0	1	0	0	0	0	0	3	0	0	0	17	2	0	8
	22	0	0	17	4	0	0	31	34	17	10	0	0	0	3	4	6	0	0	17	9	0	0	6	11	51	0
	24	2	37	5	3	14	9	5	11	15	4	4	0	4	4	0	0	0	20	0	0	5	0	29	10	0	15
POM	1	0	5	0	6	0	11	15	10	22	0	3	3	0	3	0	4	8	0	6	9	0	37	0	0	7	4
	2	0	0	12	2	2	0	33	0	0	4	0	8	7	0	7	2	0	1	0	10	5	0	0	0	0	30
	3	6	0	0	15	0	0	6	9	9	27	4	0	0	0	0	6	3	0	29	9	41	0	0	5	0	1
PRE	1	0	5	4	0	62	18	0	32	23	14	0	4	0	2	0	0	0	0	5	0	5	0	0	4	0	0
	4	0	4	4	0	0	0	0	18	23	22	17	4	7	4	5	4	4	8	13	8	6	4	0	5	5	4
	5	1	0	0	0	0	0	2	4	0	5	0	5	4	7	0	0	0	0	0	0	10	0	8	0	0	10
MUR	2	0	0	6	12	6	17	0	0	0	36	21	18	7	30	13	0	0	1	0	0	1	20	3	0	0	0
	3	32	11	0	1	0	0	2	24	0	7	11	7	29	2	3	5	1	0	0	20	17	5	0	0	0	0
	4	0	2	13	9	0	8	5	5	0	0	2	27	59	0	11	7	4	7	4	6	7	12	8	3	0	3
	5	11	4	1	4	0	4	3	0	5	0	0	42	44	7	22	4	9	7	2	19	19	13	2	0	0	0
	6	0	12	4	0	0	0	0	0	9	51	9	22	27	16	40	0	4	14	0	27	9	11	52	0	0	5
	8	0	0	11	7	0	1	3	7	4	16	1	32	33	22	5	8	1	9	6	0	0	28	4	0	0	0
	10	0	5	0	0	2	2	7	4	7	0	4	11	0	31	45	3	0	17	3	2	16	0	25	5	0	4
	13	1	0	0	21	0	0	8	31	0	0	34	5	27	10	14	22	5	5	0	28	5	12	0	0	0	0
	14	0	4	13	5	24	37	4	0	47	6	5	13	34	29	3	0	3	3	0	0	0	38	26	4	8	0
	19	0	0	0	3	3	2	0	0	0	0	0	4	5	0	8	16	0	0	24	0	15	0	7	29	9	0
	21	0	3	0	4	7	3	0	6	16	6	9	0	0	42	0	9	11	3	2	0	0	0	0	7	7	0
	23	37	5	8	0	3	1	0	11	40	0	0	5	0	0	0	4	0	0	45	51	7	0	20	1	7	0
	25	0	0	23	47	3	9	11	1	0	8	0	10	25	0	0	37	0	0	10	4	38	3	0	2	13	3
	26	0	0	2	3	0	2	0	0	2	4	10	49	6	9	0	37	6	18	37	0	4	8	10	2	5	0
	27	4	4	0	1	2	0	0	3	0	10	0	0	0	0	0	0	4	16	7	4	4	0	1	4	23	26
	29	4	4	7	0	0	8	6	0	0	6	0	11	0	0	7	5	0	0	34	2	50	6	24	2	28	2
	30	0	0	4	0	8	13	17	0	30	8	0	17	0	5	24	2	30	2	25	29	19	3	26	10	25	0
	32	1	29	0	4	0	6	0	0	0	5	0	6	0	5	6	26	0	13	24	3	32	0	25	31	6	13
	33	0	5	0	0	0	0	13	11	19	1	10	0	0	14	14	38	32	8	17	26	25	1	6	6	24	0
	34	0	0	1	0	1	18	0	1	0	5	16	0	0	0	5	0	9	0	15	18	6	4	0	17	0	18
	35	1	0	21	3	0	9	15	0	12	0	15	18	2	17	0	13	48	17	19	29	0	43	4	37	10	0
	36	8	13	0	1	19	0	2	22	1	5	28	0	0	3	0	46	14	7	6	11	8	0	0	31	10	21
	12	0	0	0	0	0	0	6	0	0	0	16	0	0	38	9	0	6	0	0	0	0	N/A	19	2	0	0
	15	4	4	5	0	4	1	4	0	0	5	5	13	0	31	27	1	10	6	0	0	13	19	N/A	8	1	0
	20	12	3	0	1	3	7	5	0	14	0	3	0	0	3	8	20	22	20	19	0	5	2	8	N/A	17	30
	28	0	0	0	1	0	0	6	12	12	0	0	0	3	7	1	4	7	4	17	7	8	0	1	17	N/A	32
	37	6	25	0	3	4	2	22	6	5	3	4	0	0	0	0	9	0	9	5	0	0	0	0	30	32	N/A
PSP	1	7	3	2	0	16	0	1	0	0	43	49	10	0	16	0	0	21	0	0	1	0	14	13	3	0	26

Table S6. Pairwise relatedness of broodstock candidates among the *Steindachneridion parahybae* individuals maintained in the germplasm bank, assessed by the ML-Relate package.

F \ M		PRJ									PRE		MUR														
		7	8	11	13	16	17	19	20	23	2	3	1	7	9	11	16	17	18	22	24	31	12	15	20	28	37
PRJ	1	3	0	1	8	0	0	9	0	0	0	2	23	0	0	20	0	0	0	0	22	12	0	0	3	11	6
	2	0	20	0	11	13	8	25	28	50	10	9	0	13	0	0	0	0	0	3	0	23	0	0	13	26	0
	3	27	40	0	1	4	0	0	0	0	0	0	0	1	15	4	0	0	0	0	8	0	0	3	5	0	10
	4	0	7	0	16	37	3	10	0	13	6	5	5	27	15	0	0	6	0	0	0	0	0	19	0	25	5
	5	39	9	33	6	16	12	11	60	43	2	0	0	0	0	0	0	0	6	45	0	0	0	0	0	0	0
	6	0	13	19	4	29	17	6	25	50	0	8	0	0	0	0	6	0	0	16	0	0	0	0	0	0	0
	9	4	9	0	24	20	1	4	50	0	21	3	0	0	0	0	4	3	0	0	0	0	0	0	0	4	0
	10	9	0	0	3	12	0	0	17	0	19	0	0	0	0	0	3	0	0	0	0	0	12	0	0	5	2
	12	0	5	14	50	28	14	0	11	4	0	1	0	0	0	0	0	0	12	19	0	0	0	0	0	0	0
	14	4	0	0	11	50	0	0	30	37	24	0	0	19	0	0	0	0	0	3	25	28	0	0	0	0	0
	15	0	28	27	23	43	22	0	29	13	0	6	0	11	0	1	6	12	0	18	0	0	0	0	0	0	5
	18	0	0	50	7	31	18	15	0	14	9	0	0	0	0	0	0	0	0	25	0	12	0	0	8	6	7
	21	6	37	0	0	76	10	0	13	17	42	8	0	10	0	0	0	0	0	0	0	0	0	33	0	0	50
	22	0	0	18	0	0	0	14	40	15	0	0	0	0	4	6	12	0	0	35	5	0	0	9	17	50	0
	24	14	12	7	0	14	11	12	24	19	3	0	0	0	0	0	0	0	50	0	0	7	0	32	23	0	25
POM	1	0	9	0	5	0	8	0	19	30	0	0	4	0	6	0	1	10	1	13	6	0	20	0	0	5	3
	2	0	0	24	0	4	0	37	0	0	9	0	0	9	0	11	0	0	0	0	6	1	0	0	0	0	31
	3	6	0	0	10	0	0	0	9	21	30	0	0	0	0	3	12	12	0	15	8	33	0	0	0	0	0
PRE	1	0	0	3	0	42	14	0	26	35	14	0	3	0	0	0	0	0	0	10	0	6	0	0	6	0	0
	4	0	1	3	0	0	0	0	17	24	15	28	12	1	0	1	0	0	9	10	10	12	0	0	5	0	0
	5	5	0	0	0	0	0	2	0	0	14	18	0	19	14	0	0	0	0	0	0	1	0	0	0	0	2
MUR	2	0	0	0	8	6	18	0	0	0	46	0	37	8	25	15	0	0	0	0	0	2	14	15	0	0	0
	3	32	13	0	9	0	0	2	33	0	0	8	0	30	6	0	9	0	0	0	30	13	14	0	0	0	0
	4	0	0	17	15	0	8	9	14	0	0	1	19	61	0	12	1	1	0	0	7	2	15	17	0	0	5
	5	20	2	0	5	0	8	18	0	0	0	0	33	38	0	13	0	0	6	0	21	13	3	1	1	0	0
	6	0	8	0	0	0	0	0	0	16	50	12	34	10	17	38	0	3	10	0	18	17	0	52	0	0	0
	8	0	0	12	9	0	8	6	1	9	12	6	12	24	26	8	0	3	4	0	0	0	33	0	0	0	0
	10	0	0	0	0	1	0	1	0	0	0	7	0	0	27	38	0	0	17	0	5	17	0	19	12	0	5
	13	11	0	0	17	0	0	0	16	0	0	23	3	12	23	10	19	0	0	0	11	0	11	0	0	0	0
	14	0	4	3	0	23	31	0	0	47	12	0	15	28	8	1	0	0	1	0	0	0	23	27	0	11	0
	19	0	0	0	0	10	7	0	0	0	0	0	5	3	0	14	21	0	0	23	0	11	0	0	36	1	0
	21	0	1	0	0	18	0	0	0	0	0	21	0	0	50	0	13	12	19	7	0	0	0	0	0	10	0
	23	30	4	17	0	10	6	0	10	28	0	0	4	0	0	0	0	0	0	15	50	0	11	41	0	0	0
	25	0	0	33	33	5	10	11	2	0	11	0	12	12	0	0	21	0	0	0	4	5	1	0	3	2	2
	26	0	0	8	0	0	0	0	0	3	9	26	50	0	14	0	40	8	15	31	6	0	19	0	0	17	0
	27	0	0	0	9	12	0	0	9	0	11	6	0	0	0	0	0	17	10	0	0	0	0	5	0	21	33
	29	1	0	16	0	0	11	7	0	0	3	0	30	0	0	2	9	0	0	30	0	18	4	21	0	18	5
	30	0	0	2	0	8	5	18	0	22	11	0	27	5	12	24	0	19	0	0	16	18	6	20	10	33	0
	32	0	6	0	3	0	5	0	0	0	0	0	5	0	9	3	18	0	13	27	0	11	0	21	27	0	0
	33	0	3	0	0	0	0	6	10	14	0	15	0	0	11	10	17	20	5	26	14	32	4	0	7	27	0
	34	0	0	6	0	8	20	0	0	2	1	20	0	0	0	8	0	20	0	23	11	0	17	5	19	0	6
	35	0	0	14	4	0	16	13	0	14	0	11	23	1	1	0	0	48	23	10	5	0	33	6	26	16	0
	36	5	9	0	8	17	0	21	9	0	4	32	0	0	5	0	41	17	8	0	1	11	0	0	35	11	25
	12	0	0	0	0	0	0	3	0	0	0	16	0	0	36	14	0	7	4	0	0	0	N/A	20	0	0	0
	15	0	0	7	0	0	0	0	0	0	0	12	23	0	32	29	0	7	11	0	0	10	20	N/A	12	1	0
	20	1	4	0	6	0	1	0	0	18	0	3	0	0	0	7	20	27	16	23	0	1	0	12	N/A	16	29
	28	0	0	0	7	0	0	8	20	26	0	0	0	1	11	0	3	8	0	13	0	0	0	1	16	N/A	17
	37	4	14	0	9	6	0	31	0	0	0	0	0	0	0	0	11	0	10	13	0	0	0	0	29	17	N/A
PSP	1	8	0	0	0	25	0	0	0	0	54	50	0	0	0	0	0	19	0	0	0	7	4	15	1	5	7
