# Supplementary material for: Genetic Diversity of an Imperiled Neotropical Catfish and Recommendations for Its Restoration
Source: Front Genet. 2017 Dec 12;8:196. doi: 10.3389/fgene.2017.00196 (PMC5732928; doi:10.3389/fgene.2017.00196)
Supplement: Supplementary file 2 [file Supplementary_Material_Image.pdf]

SUPPLEMENTARY MATERIAL - IMAGES

Genetic diversity of an imperiled Neotropical catfish and recommendations for its restoration

Fernando Stopato da Fonseca, Rodrigo Rodrigues Domingues, Eric M. Hallerman and Alexandre Wagner Silva Hilsdorf

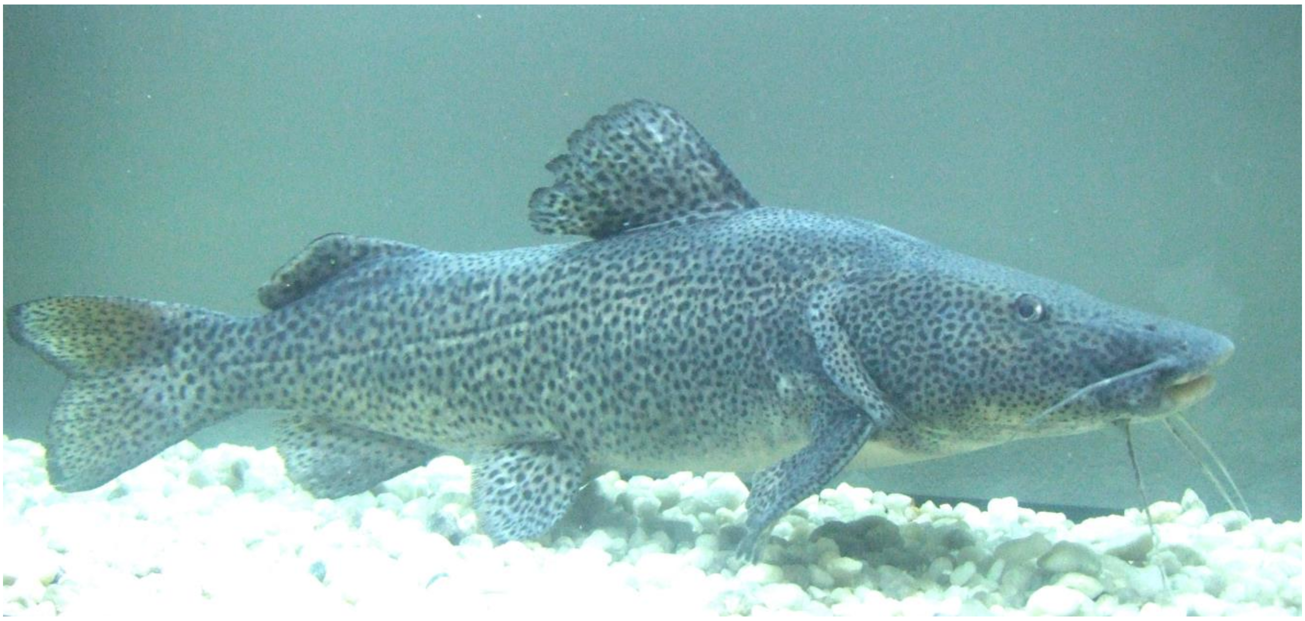

Image S1. Adult *Steindachneridion parahybae* (surubim-do-paraíba). Photo: Companhia Energética de São Paulo (CESP) photo repository.

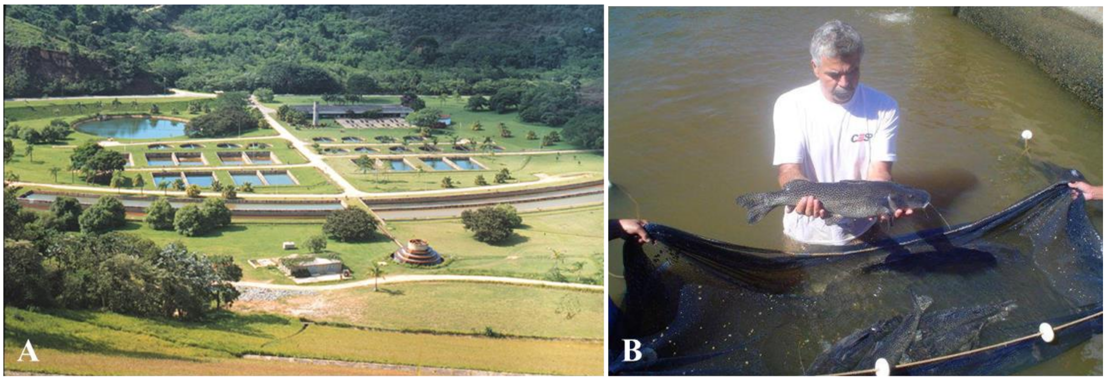

Image S2. (A) Hatchery facilities of the Companhia Enérgica de São Paulo (CESP) used as an *ex-situ* germplasm bank. **(B)** A *Steindachneridion parahybae* wild broodstock used in the restocking program. Photos: (A) Alexandre W.S. Hilsdorf; (B) Danilo Caneppele.
